# Construction of a complete set of alien chromosome addition lines from *Gossypium australe* in *Gossypium hirsutum*: morphological, cytological, and genotypic characterization

**DOI:** 10.1007/s00122-014-2283-1

**Published:** 2014-02-20

**Authors:** Yu Chen, Yingying Wang, Kai Wang, Xiefei Zhu, Wangzhen Guo, Tianzhen Zhang, Baoliang Zhou

**Affiliations:** State Key Laboratory of Crop Genetics and Germplasm Enhancement, MOE Hybrid Cotton R&D Engineering Research Center, Nanjing Agricultural University, Nanjing, 210095 Jiangsu People’s Republic of China

## Abstract

*****Key message***:**

**We report the first complete set of alien addition lines of** ***G. hirsutum*** **. The characterized lines can be used to introduce valuable traits from** ***G. australe*** **into cultivated cotton.**

**Abstract:**

*Gossypium australe* is a diploid wild cotton species (2*n* = 26, GG) native to Australia that possesses valuable characteristics unavailable in the cultivated cotton gene pool, such as delayed pigment gland morphogenesis in the seed and resistances to pests and diseases. However, it is very difficult to directly transfer favorable traits into cultivated cotton through conventional gene recombination due to the absence of pairing and crossover between chromosomes of *G. australe* and *Gossypium hirsutum* (2*n* = 52, AADD). To enhance the transfer of favorable genes from wild species into cultivated cotton, we developed a set of *hirsutum*–*australe* monosomic alien chromosome addition lines (MAAL) using a combination of morphological survey, microsatellite marker-assisted selection, and molecular cytogenetic analysis. The amphidiploid (2*n* = 78, AADDGG) of *G. australe* and *G. hirsutum* was consecutively backcrossed with upland cotton to develop alien addition lines of individual *G. australe* chromosomes in *G. hirsutum*. From these backcross progeny, we generated the first complete set of chromosome addition lines in cotton; 11 of 13 lines are monosomic additions, and chromosomes 7G^a^ and 13G^a^ are multiple additions. MAALs of 1G^a^ and 11G^a^ were the first to be isolated. The chromosome addition lines can be employed as bridges for the transfer of desired genes from *G. australe* into *G. hirsutum*, as well as for gene assignment, isolation of chromosome-specific probes, flow sorting and microdissection of chromosome, development of chromosome-specific ‘‘paints’’ for fluorochrome-labeled DNA fragments, physical mapping, and selective isolation and mapping of cDNAs for a particular *G. australe* chromosome.

**Electronic supplementary material:**

The online version of this article (doi:10.1007/s00122-014-2283-1) contains supplementary material, which is available to authorized users.

## Introduction

Cotton, *Gossypium* spp., is the most important natural textile fiber, the second best potential source of plant protein and the fifth largest oilseed crop in the world (Bi et al. 1999a, b). The genus *Gossypium* consists of approximately 45 diploid (A–G and K genomes, 2*n* = 2*x* = 26) and five tetraploid species (AD genome, 2*n* = 4*x* = 52) (Fryxell 1992). All of these species have been classified into primary, secondary, and tertiary germplasm pools (Stewart 1995) on the basis of the relative genetic accessibility and utility of species to cotton improvement efforts (Harlan and de Wart 1971), being a valuable reservoir of agronomically useful genes and genes for resistance to pests and diseases (Endrizzi et al. 1985; Stewart 1995).

*G. australe* F. Mueller, a wild diploid cotton species (2*n* = 2*x* = 26, GG) native to Australia, possesses numerous economically valuable characteristics such as delayed pigment gland morphogenesis, which would be conducive to production of seeds with very low levels of gossypol as a potential source of food and feed for both human and animal consumption, resistance to pest insects (aphids and mites) and diseases (Fusarium and *Verticillium* wilts), and tolerance to abiotic stresses (drought), that would be useful if transferred into the most important tetraploid cultivated species, *G. hirsutum* L. (2*n* = 4*x* = 52, AADD). *G. australe*, however, belongs to the tertiary gene pool of *Gossypium*. These species present the greatest challenge to breeders for utilization in cotton improvement, because differences in chromosome number and structure restrict homoeologous chromosome recombination and limit introgression across genomes due to the failure of corresponding chromosomes to pair at meiosis in the interspecific hybrid with *G. hirsutum*. Thus, the transfer of genes from *G. australe* directly into *G. hirsutum* cannot be accomplished by conventional breeding methods such as backcrossing. One strategy is to produce monosomic alien addition lines (MAALs, 2*n* = 4*x* = 52 + II) by repeated backcrossing to an allohexaploid (2*n* = 6*x* = 78) of the *G. hirsutum* × *G. australe* F_1 _(2*n* = 3*x* = 39) by chromosome doubling following colchicine treatment, which can be used as a bridge to transfer desired genes from *G. australe* into *G. hirsutum* (Stewart, 1995). Over the past two decades, MAALs have been widely available in wheat (Friebe et al. 2000; Kishii et al. 2004; Wang et al. 2001; Kong et al. 2008), rice (Multani et al. 2003), potato (Ali et al. 2001), cucumber (Chen et al. 2004), tobacco (Chen et al. 2002), oat (Kynast et al. 2001), sugar beet (Reamon-Ramos and Wricke 1992; Gao et al. 2001), and rapeseed (Srinivasan et al. 1998; Budahn et al. 2008). The system has been used in numerous studies, such as chromosome pairing (Cifuentes and Benavente 2009; Molnár and Molnár-Láng 2010), recombination (Ji and Chetelat 2003; Pertuzé et al. 2003), gene transfer (Peterka et al. 2004; Fu et al. 2012; Chen et al. 1992), gene mapping (Geleta et al. 2012; Chen et al. 1992), gene tagging, genome structure, evolution, microdissection, and microcloning for chromosome-specific library construction (Shim et al. 2010; Kynast et al. 2004; Fang et al. 2004; Jiang et al. 2005; Bento et al. 2010). The application of molecular biological techniques using such stocks led to development of the field of molecular cytogenetics, allowing specific DNA sequences to be mapped to a physical chromosomal location.

In cotton, several previous reports on efforts to isolate alien addition lines of diploid *Gossypium* species in *G. hirsutum* were based mainly on classical cytogenetic analyses combined with morphological observation (Hau 1981; Rooney and Stelly 1991; Mergeai 1992). Due to the unreliability and time-consuming nature of these techniques, however, there was no complete set of diploid species MAALs in *G. hirsutum* reported by the end of twentieth century. With the advent of molecular genetic markers and molecular cytogenetic techniques such as genomic in situ hybridization (GISH) in cotton, and the construction of cotton molecular genetic maps, cotton researchers have new tools to identify and characterize MAALs for transferring wild traits of interest. The above techniques have facilitated the isolation of MAALs. Ahoton et al. (2003) isolated six of the possible 13 MAALs carrying *G. australe* chromosomes in a *G. hirsutum* background. Sarr et al. (2011) also identified five new MAALs of *G. australe* in *G. hirsutum* using simple sequence repeat (SSR) and GISH analysis based on the results of Ahoton et al. (2003). Zhou et al. (2004) also isolated two MAALs of *G. somalense* in *G. hirsutum* using random amplified polymorphic DNA (RAPD) and classical cytogenetic analysis.

In order to develop a complete set of monosomic alien chromosome addition lines from *G. australe* into *G. hirsutum*, we backcrossed the interspecific hexaploid hybrid of *G. hirsutum* × *G. australe* to *G. hirsutum*, and pentaploid plants (i.e., BC_1_) were obtained for a subsequent backcross with *G. hirsutum*. For the BC_2_, BC_3_, BC_4_, and BC_5_ progeny, GISH was employed to identify chromosome(s) from *G. austral*. Furthermore, a set of *G. australe*-specific SSR markers were developed and used to identify *G. australe* chromosome(s) that were present in the *G. hirsutum* background. As a result of this work, eleven MAALs, one double monosomic alien addition line (DMAAL), and one triple monosomic alien addition line (TMAAL) were identified and characterized.

## Materials and methods

### Plant materials

**Fig. 1:**
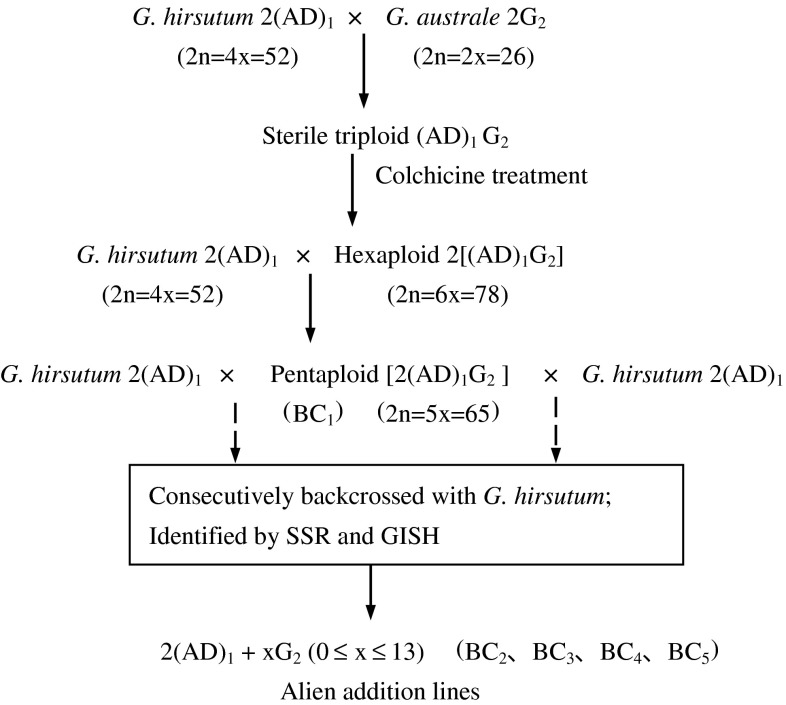
Scheme for the development of alien chromosome addition lines of *G. australe* in *G. hirsutum*

An allohexaploid cotton line was obtained from the chromosome-doubled triploid F_1_ of the cross *G. hirsutum* × *G. australe*. The allohexaploid (kindly provided by Dr. Curt Brubaker; Brubaker and Brown 2003) used as the paternal parent was directly crossed with *G. hirsutum* acc. TM-1, and seven pentaploid seeds were obtained in 2007; the six pentaploid plants obtained were grown at Jiangpu Breeding Station of Nanjing Agricultural University (JBS/NAU). When they flowered, all six pentaploid plants were backcrossed with TM-1 at the Pailou Experimental Station of Nanjing Agricultural University (PES/NAU); the plant hormone GA_3_ was applied to the flower base for protection from shedding when crossing. All cottonseeds obtained were sown in nursery pots and the seedlings were transplanted into clay pots in the field. In winter, all plants were moved into a glasshouse at PES/NAU. The scheme for developing the monosomic alien addition lines is shown in Fig. 1.

### DNA extraction and SSR primer pair screening

Total genomic DNA was extracted from young leaves of the two parents, *G. hirsutum* and *G. australe*, the interspecific hexaploid, and individuals of the BC_1_, BC_2_, BC_3_, BC_4_, and BC_5_ generations as described by Paterson et al. (1993) with some modifications.

Based on the high-density tetraploid cotton linkage map constructed in our institute (Guo et al. 2007), 2,168 microsatellite (simple sequence repeat, SSR) primer pairs were selected to screen for polymorphisms between the two parental species, *G. hirsutum* and *G. australe*. All SSR primer information used in this work can be downloaded from http://www.cottonmarker.org. SSR-PCR amplifications were performed using a Peltier Thermal Cycler-EDC-810 (Eastwin, Hong Kong), and electrophoresis of the products was performed as described by Zhang et al. (2000, 2002). *G. australe*-specific marker primers were further employed to identify chromosomes from *G. australe*.

### MAAL nomenclature

Since previous studies have mapped numerous SSR markers onto *G. hirsutum* chromosomes (Guo et al. 2007), and these SSR markers are cross-transferable in *Gossypium* species (Guo et al. 2006), they can be used to reveal the homoeologous relationships between *G. australe* and *G. hirsutum* chromosomes, as validated by Ahoton et al. (2003). In order to employ a consistent nomenclature as is used for wheat chromosomes (Sears 1954), the thirteen chromosomes of *G. australe* will be designated 1G^a^–13G^a^, based on SSR marker distributions on chromosomes in the D_t_-subgenome of tetraploid map. There are very few chromosomal structural changes in the D_t_-subgenome of cultivated tetraploid cotton. In particular, no chromosome translocations have occurred throughout evolution (Gerstel and Sarvella 1956); thus, the chromosome codes 1–13 in *G. australe* should correspond to the homoeologous chromosome designations in the D_t_-subgenome of tetraploid cotton. The thirteen MAALs will be named MAAL-1G^a^ to MAAL-13G^a^, G^a^ indicates that *G. australe* belongs to the G-genome species in *Gossypium*, and the superscript letter ‘a*’* refers to the initial letter in the species name *australe*.

### Chromosome preparation

Root tips from each individual in every BC generation were harvested from germinated seeds and pretreated in 25 μg/mL cycloheximide at room temperature for 2 h to accumulate metaphase cells. Root tips were then fixed in a solution of ethanol:acetic acid (3:1 v/v). After fixation, root tips were rinsed in distilled water and then macerated in 4 % cellulase and 1 % pectinase at 37 °C for 1.5 h and squashed in a drop of 45 % acetic acid. Slides with more than 20 good images of well-spread somatic chromosomes at metaphase were prepared and then stored at −70 °C until use. Two slides were prepared and about 20 cells were examined for each individual. The coverslips were removed from the slides, and the root tips were dehydrated through an ethanol series (70, 90, and 100 %; 5 min each) prior to use in fluorescence in situ hybridization (FISH). Meiotic chromosome spreads were prepared as per Wang et al. (2006) with several modifications. Upon the removal of the calyx and corolla, floral buds from each individual of MAALs were fixed in ethanol–acetic acid (3:1) fixative for 2–24 h at 4 °C. Next, the buds were screened for metaphase I, and several anthers from the selected bud were placed on an ethanol-washed glass slide with a drop of 45 % acetic acid (v/v), freed of debris, and squashed.

### Fluorescence in situ hybridization (FISH)

Genomic DNA extracted from *G. australe* was labeled with digoxigenin-11-dUTP and biotin-16-dUTP (Roche Diagnostics, Mannheim, Germany) by nick translation. The sizes of the labeled DNA probe fragments were between 200 and 500 bp. Fluorescence in situ hybridization was carried out as described by Wang et al. (2006) with some modifications. Chromosomal DNA was denatured by placing the slides in 50 mL 70 % formamide, 2 × SSC at 72 °C for 2.5 min, and then immediately dehydrating them in an ethanol series at −20 °C followed by air-drying. Fifteen microliters of a mixture containing 25–50 ng labeled DNA, 50 % (w/v) dextran sulfate, 10-μg sheared salmon sperm DNA, an appropriate amount of sheared cotton DNA as blocking DNA (probe: blocking DNA = 1:100), and 1.5 μL 20 × SSC, was denatured at 97 °C for 10 min, chilled on ice, annealed at 37 °C for 1 h, and then applied to a dry slide. Following overnight incubation at 37 °C, the coverslips were removed and the slides were washed at increased stringency by rinsing at 43 °C in: 2 × SSC for 5 min (twice); 2 × SSC, 60 % formamide for 13 min; twice more in 2 × SSC for 5 min; and finally in 1× PBS for 5 min. Probes were detected with 20 μg/mL rhodamine-conjugated anti-digoxigenin antibody and avidin–fluorescein (Roche Diagnostics). Slides were stained in 4′,6-diamidino-2-phenylindole (DAPI) (Roche Diagnostics) for 10 min at room temperature, and anti-fade (Vector, USA) was applied under the coverslip. Slides were examined and more than 20 images of well-spread somatic chromosomes at metaphase stage were obtained for each individual using an Olympus BX51 fluorescence microscope. Chromosome and FISH signal images were captured using an Evolution VF CCD camera (Media Cybernetics, Bethesda, MD, USA) and merged using Image-Pro Express software (Media Cybernetics, Bethesda, MD, USA).

### Evaluation of traits

The seed index was determined by weighing 100 cottonseeds collected from open bolls of TM-1 (control) and MAAL-4G^a^. For the measurement of leaf chlorophyll content of TM-1 (control) and MAAL-8G^a^, the youngest fully expanded leaves were collected to extract chlorophyll as described by Bao and Leng (2005). The chlorophyll *a* and chlorophyll *b* contents were measured as described by Wang and Zhang (2012) with three experimental and three biological replicates. The shape and size of the bolls were observed at 40 days post-anthesis. Fully expanded leaves from the same position on the MAALs and the control (TM-1) plants were investigated for shape and size. Floral morphological traits were observed at the time of flowering.

## Results

### Development of set of putative *G. australe* chromosome-specific SSR primers pairs

**Fig. 2:**
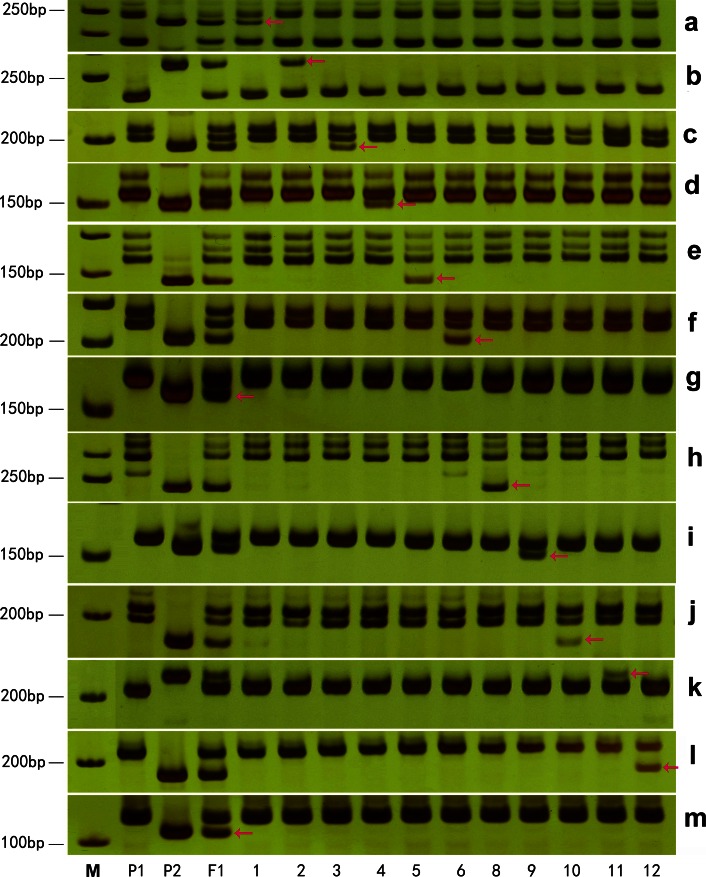
A set of *G. australe*-*specific* SSR markers were used to identify individual alien chromosomes of *G. australe* in *G. hirsutum*. **a**–**m** The *G. australe*-*specific* amplicons were obtained using 13 individual chromosome-specific primer pairs for markers; NAU5138, NAU6692, NAU805, NAU2120, Cgr5510, dPL0811, dPL0048, NAU6616, NAU3763, NAU4973, NAU2141, dPL0379, and BNL193. The chromosomes correspond to D_1_–D_13_ in cultivated tetraploid cotton. *P1* *G. hirsutum,* *P2* *G. australe,* *F*_1_ the hexaploid of *G. hirsutum* and *G. australe*; *1–6* and *8–12* show that each of these plants possesses a single different individual chromosome from *G. australe*, corresponding to 1G^a^ to 6G^a^, and 8G^a^ to 12G^a^, but not 7G^a^ or 13G^a^. *M* molecular size marker (50 bp ladder). *Arrows* (*red*) indicate chromosome-specific markers for *G. australe*

We used a total of 2,168 SSR primer pairs/combinations selected from the linkage maps of the *G. hirsutum* and *G. barbadense* genomes constructed in our institute (Guo et al. 2007) to screen for genetic polymorphisms between *G. hirsutum* and *G. australe*. The results indicated that approximately 66.2 % (1435/2168) of the SSRs detected polymorphisms between these two species. It is well known that translocations have occurred between chromosomes in the At-subgenome of the tetraploids, as well as At-subgenome and A-genome species (Gerstel and Sarvella 1956; Guo et al. 2007), while very few incidences of chromosomal structural changes have occurred in the D_t_-subgenome between genomes during tetraploid formation. Hence, we selected and used only unambiguously polymorphic SSR primers in the D_t_-subgenome of the tetraploids. In this study, after removing markers clustered on the chromosomes, a total of 160 *G. australe*-specific marker alleles that were almost evenly distributed on each D_t_-subgenome chromosome were obtained to monitor chromosomes of *G. australe* in *G. hirsutum*. For each chromosome, there were 10–18 SSR primer pairs/combinations obtained based on the homoeological relationships that exist between *G. australe* and *G. hirsutum* chromosomes. These SSR markers covered from 80.5 to 97.3 % of each *G. australe* chromosome with a density of 6.7–15.0 cM (Table 1; Fig. 2, Fig. 1S). The *G. australe*-specific SSR markers have been validated in the development of *hirsutum*–*australe* alien chromosome addition lines for monitoring the numbers and identities of chromosomes of *G. australe* in combination with GISH.

**Table 1:** SSR primers used to screen for *G. australe* chromosomes in the alien addition lines

Chr	1G	2G	3G	4G	5G	6G	7G	8G	9G	10G	11G	12G	13G
	NAU458-230	NAU3733-490	shin-250	NAU6992-200	NAU3095-210	BNL3359-200	NAU5325-180	BNL2597-220	BNL686-150	**MUSS266-180**	NAU5461-210	NAU3032-390	**NAU3685-120**
	NAU3346-280	JESPR-156-90	TMK08-190	BNL4030-130	NAU4907-350	BNL2569-680	BNL1694-240	NAU3010-160	NAU2739-190	STV031-160	dPL0522-195	NAU3862-270	**BNL193-100**
	NAU3714-170	STV030-135	BNL3955-150	JESPR-65-130	NAU2232-450	BNL3103-190	BNL1604-110	NAU5130-330	NAU6701-390	NAU2549-260	BNL1034-200	BNL3537-170	**NAU7471-160**
	NAU3056-630	NAU4009-160	NAU5333-150	dPL0390-230	JESPR236-400	NAU2397-240	TMB09-280	dPL0862-220	NAU6517-300	NAU6720-150	Gh508-200	NAU4914-200	**dPL0286-220**
	NAU5172-180	NAU2633-240	JESPR101-160	NAU3508-250	BNL3535-145	dc40121-180	cot030-200	shin0384-200	NAU6751-135	NAU4973-170	**Gh83-140**	NAU1558-380	cgr5390-340
	MUCS322-450	NAU7342-270	NAU1028-120	MUCS271-360	**dPL0137-140**	BNL3806-160	NAU2620-320	cgr5503-120	NAU5508-160	Gh424-95	NAU5436-370	BNL341-160	BNL1079-200
	BNL2646-120	**Gh51-130** ^**d**^	NAU6129-280	BNL358-250	BNL390-210	Gh537-160	NAU4956-280	Gh573-145	NAU2709-200	**TMF09-240**	BNL2632-340	NAU7594-500	BNL3280-200
	NAU4073-180	NAU6692-270	JESPR-304-140	cgr5566-155	**NAU6406-230**	JESPR229-115	cgr5149-120	cgr5202-150	JESPR208-160	NAU0922-220	cgr5233-430	cgr5787-120	NAU3203-160
	NAU2814-230	NAU4022-200	NAU2859-270	NAU3437-250	**NAU5486-270**	dPL0702-210	NAU2597-190	NAU3904-240	JESPR110-170	NAU1280-130	Gh434-150	NAU3442-320	BNL2652-280
	cgr5309-140	NAU3573-800	NAU2898-230	NAU7290-200	Gh109-120	NAU2641-180	cgr5181-120	JESPR291-170	NAU3414-240	NAU1169-300	cgr5148-140	NAU1119-220	NAU6724-370
		dc40046-240		JESPR-220-150	cgr5117-130	MUSS519-160			cgr6572-330	STV031-370	**CIR414-160**	BNL2578-250	dPL0864-200
		**NAU6728-330**			Gh229-130	NAU3502-240			NAU3414-250		NAU2602-310	NAU4925-600	BNL2571-360
					cgr5510-150				NAU864-170		Gh132-170	NAU3271-200	NAU3861-450
					cgr5803-120						NAU486-230		NAU3843-280
					cgr6240-240						NAU429-220		
					**Gh182-190**						NAU6658-180		
					JESPR122-320								
					**NAU3631-270**								
Total	10	12	10	11	18	12	10	10	13	11	16	13	14
Position	0.0–107.3	5.6–110.4	14.0–119.5	3.5–105.9	10.9–185.4	8.5–123.9	2.4–121.1	0.0–149.9	0.0–143.9	0–112.4	5.8–157.7	15.3–126.2	8.8–103.1
Mean density^a^	10.7	8.7	10.6	9.3	9.7	9.6	11.9	15	11.1	10.2	9.5	8.5	6.7
GDC (cM)^b^	107.3	104.8	105.5	102.4	174.5	115.4	118.7	149.9	143.9	112.4	151.9	110.9	94.3
PCC (%)^c^	85.3	93.5	83.6	80.2	91.9	83.7	91.7	90.3	95.2	97.3	83.9	83.0	80.5

^a ^Genetic distance (cM) between two adjacent markers on a chromosome

^b^ *GDC* genetic distance coverage (cM)

^c^ *PCC* percentage of chromosome covered by markers (%)

^d ^Boldface type indicates the molecular markers that are deleted in the MAALs

### Transmission rates of alien chromosomes of *G. australe* in *G. hirsutum*

Using the pentaploid F_1_ of *G. hirsutum* × *G. australe* (2*n* = AADDGG = 78) × *G. hirsutum* as the maternal parent consecutively backcrossed with *G. hirsutum*, we found that different *G. australe* chromosomes had diverse transmission rates during backcrossing into the upland cotton background, and that the number of alien chromosomes drastically decreased in successive backcross generations. These results are described in detail as follows: the BC_2_ population consisted of 63 individuals, and the number of alien chromosomes transmitted from the BC_1_ (pentaploid) ranged between 1 and 10. Most plants (85.71 %) carried 1–6 alien chromosomes, and only 14.29 % of plants carried 7–10 alien chromosomes. The alien chromosome with the highest incidence was 10G^a^, which was in 84.13 % of the BC_2_ progeny, followed by 8G^a^ at 42.86 %; 3G^a^, 5G^a^, and 12G^a^ at 38.10 %; 2G^a^ and 4G^a^ at 31.75 %; 11G^a^ at 23.81 %; and 1G^a^ and 6G^a^ at 20.63 %. The lowest incidence was 11.11 % for both 7G^a^ and 9G^a^. The first four isolated MAALs carried *G. australe* chromosomes 2G^a^, 5G^a^, 10G^a^, and 11G^a^. The remaining 55 plants carried multiple alien chromosomes (Table 1S).

The above multiple alien chromosome addition lines in the BC_2_ generation were further backcrossed to *G. hirsutum* (TM-1), and a total of 164 plants were characterized by PCR and GISH. Of these, 17 (10.37 %) plants had no alien chromosomes. The number of alien chromosomes transmitted from the BC_2_ were 1–5. Most plants (40.24 %) carried only one alien chromosome, followed by two (21.95 %), three (12.20 %), four (12.20 %), and five (3.05 %) *G. australe* chromosomes. The highest incidence of an alien chromosome was 85.71 % for 10G^a^ (similar to transmission from the BC_1_), followed by 27.21 % for 12G^a^. Transmission of the other chromosomes was drastically reduced to <20 %: 19.73 % for 8G^a^, 19.05 % for 3G^a^, and 12.24 % for 6G^a^; transmission was <10 % for 1G^a^, 2G^a^, 4G^a^, 5G^a^, 7G^a^, 9G^a^, 11G^a^, and 13G^a^. In the BC_3_, we isolated five new MAALs for 1G^a^, 3G^a^, 6G^a^, 8G^a^, and 12G^a^. MAAL-10G^a^ was isolated again with a rate as high as 77.27 %. The others had a very low rate of isolation (Table 2S). The remaining 81 plants were multiple alien addition lines.

The above 81 plants from the BC_3_ generation were further backcrossed with *G. hirsutum* (TM-1 as paternal parent), and a total of 237 progeny plants were characterized by PCR and GISH. Of these, 18 (7.59 %) plants had no alien chromosomes. The numbers of alien chromosomes transmitted from the BC_3_ were 1–4. The majority of plants (57.38 %) carried only one alien chromosome, followed by two (27.43 %), three (6.75 %), and four (0.84 %) chromosomes. The highest incidence for an alien chromosome was 91.32 %, again for 10G^a^, followed by 23.74 % for 12G^a^ and 13.24 % for 3G^a^. Transmission of the other chromosomes decreased drastically to <5 %: 1.83 % for 2G^a^ and 4G^a^, 1.37 % for 5G^a^ and 9G^a^, 4.11 % for 6G^a^ and 13G^a^, and 4.57 % for 8G^a^; there were no plants carrying 1G^a^, 7G^a^, or 11G^a^. In the BC_4_ generation, we isolated only one new MAAL, for 9G^a^, at the rate of 0.74 %. MAAL-10G^a^ was isolated again with a rate as high as 89.71 %. The other chromosomes had a very low rate of isolation (Table 3S). The remaining 83 plants were multiple addition lines.

The 83 plants of the BC_4_ generation were again backcrossed to *G. hirsutum* (TM-1 as paternal parent), and 32 plants in total were characterized by PCR and GISH. Of these, 15 (46.88 %) plants had no alien chromosomes. The numbers of alien chromosomes transmitted from the BC_4_ were 1–4. The majority of plants (34.38 %) carried only one alien chromosome, followed by two (15.63 %) and three (3.13 %) chromosomes. The highest incidence for an alien chromosome was 82.35 %, again for 10G^a^ as in the preceding BC generations, followed by 35.29 % for 4G^a^, 11.76 % for 5G^a^, and 5.88 % for both 3G^a^ and 13G^a^. In the BC_5_ generation, only one new MAAL, for chromosome 4G^a^, was isolated at the rate of 9.09 %. This was the same as the rates for 3G^a^ and 5G^a^, with 10G^a^ transmitted at the highest rate of 72.73 % (Table 4S).

In total, the number of progeny in all backcross generations (BC_2_ to BC_5_) consisted of 496 individuals, and the numbers of alien chromosomes carried by these plants ranged from 1 to 10. Most of the plants (44.56 %) carried only a single alien chromosome, followed by two (23.19 %), and three or four (combined 16.13 %) chromosomes. A few plants (6.05 %) carried between 5 and 10 alien chromosomes. The highest incidence for any alien chromosome was for 10G^a^ with a rate as high as 79.03 %, followed by 12G^a^ at 22.18 %; 3G^a^ at 16.53 %, 8G^a^ at 13.31 %, 6G^a^ at 8.06 %, 5G^a^ at 7.86 %, 2G^a^ and 4G^a^ both at 7.66 %; 13G^a^ at 6.65 %, 1G^a^ at 3.63 %, 9G^a^ and 11G^a^ both at 3.43 %, and 7G^a^ with the lowest rate of 2.02 %. Of these, with the exception of 7G^a^ and 13G^a^ being multiple addition lines, we isolated 11 monosomic addition lines for *G. hirsutum*. The relative order of occurrence of monosomic chromosome additions isolated, from high to low, was 10G^a^, 8G^a^, 3G^a^, 6G^a^, 5G^a^, 12G^a^, 2G^a^, 11G^a^, 1G^a^, 4G^a^, and 9G^a^, (82.81, 5.43, 3.17, 2.26, 1.81, 1.36, 0.90, 0.90, 0.45, 0.45, and 0.45 %, respectively) (Table 2).

**Table 2:** Incidence of alien (*G. australe*) chromosomes in *G. hirsutum* × *G. australe* in the BC_2_ to BC_5_ generations

Chromosome number	1G	2G	3G	4G	5G	6G	7G	8G	9G	10G	11G	12G	13G	No. of individuals
52	0	0	0	0	0	0	0	0	0	0	0	0	0	50
52 + 1	1	2	7	1	4	5	0	12	1	183	2	3	0	221
52 + 2	0	5	23	11	5	8	0	12	4	102	1	54	5	115
52 + 3	2	5	22	5	7	9	1	11	3	46	1	23	9	48
52 + 4	7	9	11	7	6	9	2	10	3	32	2	18	11	32
52 + 5	0	5	8	5	5	3	3	8	1	13	2	3	3	13
52 + 6	2	5	3	3	5	1	3	5	2	7	5	5	2	8
52 + 7	2	4	3	1	3	3	1	4	0	4	2	1	0	4
52 + 8	2	1	3	3	3	1	0	3	1	3	1	1	2	3
52 + 9	1	1	1	1	0	0	0	1	1	1	1	1	0	1
52 + 10	1	1	1	1	1	1	0	0	1	1	0	1	1	1
Sum	18	38	82	38	39	40	10	66	17	392	17	110	33	496
Incidence (%)	3.63	7.66	16.53	7.66	7.86	8.06	2.02	13.31	3.43	79.03	3.43	22.18	6.65	
Monosomic addition (%)	0.45	0.90	3.17	0.45	1.81	2.26	0.00	5.43	0.45	82.81	0.90	1.36	0.00	

### Toward development of a complete set of MAALs

**Fig. 3:**
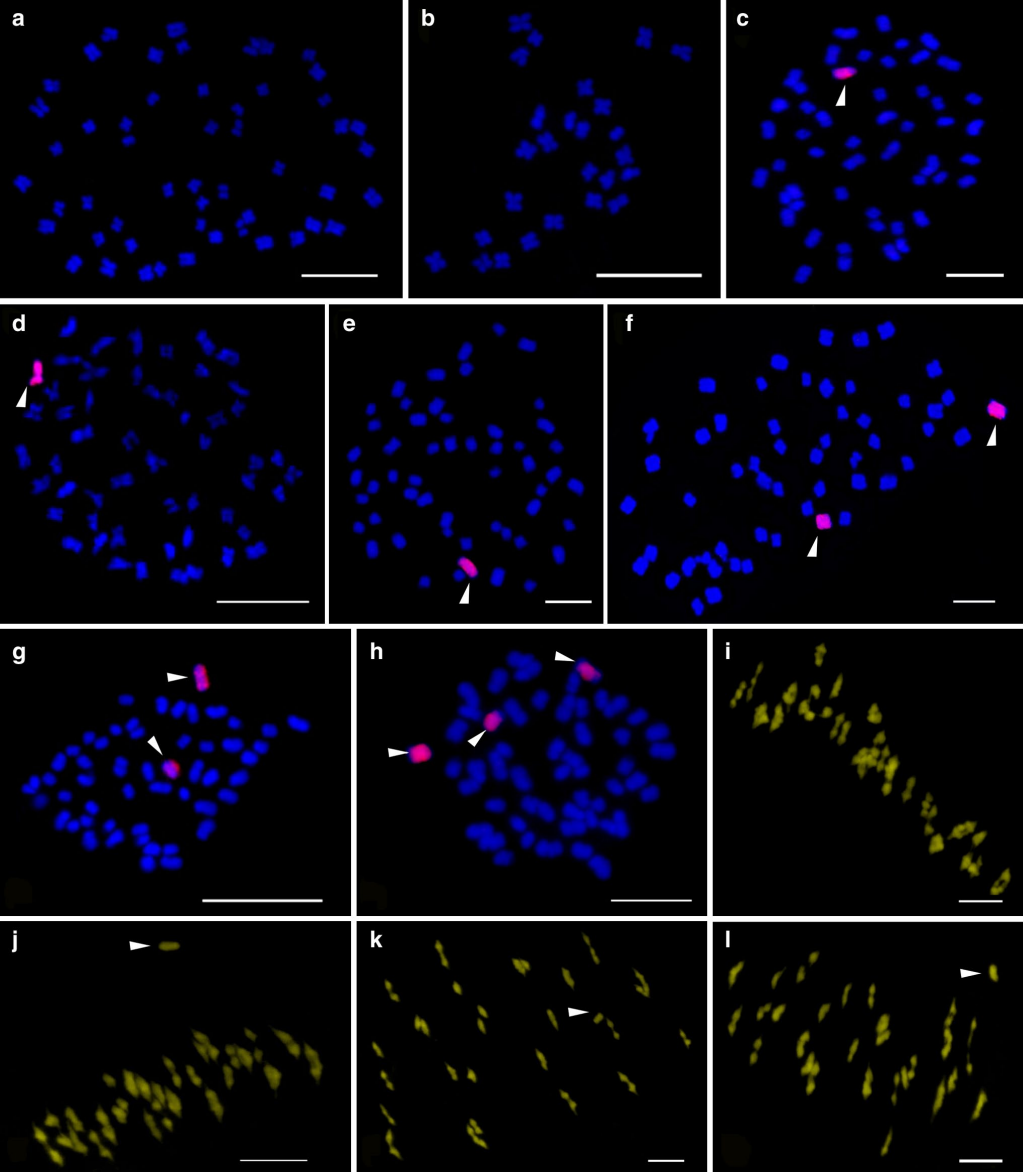
**a**–**l** Genomic in situ hybridization of the putative alien chromosomes of *G. australe* in the *G. hirsutum* background shows the chromosomal constitutions. **a** Mitotic chromosome spread of the 52 chromosomes of *G. hirsutum*. **b** Mitotic chromosome spread of the 26 chromosomes of *G. australe*. **c**, **d** and **e** Mitotic chromosome spread showing the 52 *G. hirsutum* (*blue*) chromosomes and individual chromosomes 3G^a^, 6G^a^, and 10G^a^ of *G. australe* (*red*, *white* *arrowhead*), respectively. **f**, **g** and **h** Mitotic chromosome spread showing the 52 *G. hirsutum* (*blue*) chromosomes and two (2G^a^, 10G^a^), two (10G^a^, 13G^a^) and three (7G^a^, 8G^a^, 10G^a^) chromosomes of *G. australe* (*red*, *white arrowhead*), respectively. **i** Meiotic chromosome spread showing 26 bivalents of *G. hirsutum*. **j**, **k** and **l** Meiotic chromosome spread showing 26 bivalents of *G. hirsutum* plus 1 univalent of chromosomes 5G^a^, 6G^a^, and 10G^a^ from *G. australe* (*white* *arrowhead*), respectively. *Scale bar* 3 μm

We characterized a total of 496 plants using the GISH technique and 160 SSR markers to identify chromosomes of *G. australe* in *G. hirsutum* during the isolation of a set of MAALs (Table 2, Fig. 3a–l). Our results indicated that the majority of plants (221) carried one chromosome from *G. australe*, 115 carried two, and 48 plants carried three alien chromosomes. Among the 221 MAALs identified by SSR markers distributed on the homoeologous chromosomes in the D_t_-subgenome of the tetraploids, the vast majority of the plants were MAAL-10G^a^ (183 plants), followed by MAAL-8G^a^ (12), MAAL-3G^a^ (3), MAAL-6G^a^ (5), MAAL-5G^a^ (4), MAAL-12G^a^ (3), MAAL-2G^a^ and MAAL-11G^a^ (2 plants each), and MAAL-1G^a^, MAAL-4G^a^ and MAAL-9G^a^ (1 plant each). There were no MAALs isolated for chromosomes 7G^a^ and 13G^a^ but double monosomic addition lines for chromosomes 13G^a^ with 5G^a^, and triple monosomic addition lines for chromosomes 7G^a^ with 8G^a^ and 10G^a^ were identified in *G. hirsutum*.

In conclusion, GISH analysis and SSR markers distributed on the homoeologous chromosomes of the D_t_-subgenome of tetraploid cotton enabled the isolation of the first complete set of alien addition lines of *G. australe* chromosomes in *G. hirsutum*. Eleven of these lines are monosomic additions, while chromosomes 7G^a^ and 13G^a^ are in multiple addition lines. The MAALs for 1G^a^ and 11G^a^ are the first to be reported for these chromosomes.

### Transmission rates of chromosome 10G^a^ in *G. hirsutum* through the male and female gametes and in different generations

In this study, interestingly, we found that chromosome 10G^a^ from *G. australe* showed a transmission rate of >90 % in *G. hirsutum*. To determine which gamete transmitted 10G^a^, the 10G^a^ chromosome addition lines were reciprocally crossed with TM-1. The results indicated that the 10G^a^ chromosome was mainly transmitted through the female gamete, and its transmission rate was as high as 96.5 %, while the transmission rate was only 5.7 % through the male gamete (Table 3). Theoretically, there would be 5.5 % disomic 10G^a^ addition lines in the self-pollinated progeny of monosomic 10G^a^ addition lines. In fact, to date, we have been unable to isolate any disomic 10G^a^ chromosome addition lines from self-pollinating the monosomic 10G^a^ addition lines.

**Table 3:** Transmission rates of alien chromosome 10G^a^ from *G. australe* through the female and male gametes in *G. hirsutum,* respectively

Cross	No. of plants		Total
	2*n* (%)	2*n* + 1 (%)	
(2*n* + 1) × 2*n* ^a^	4 (3.5)	109 (96.5)	113
2*n* ^a^ × (2*n* + 1)	50 (94.3)	3 (5.7)	53

^a^2*n* denotes the recurrent parent, *G. hirsutum* acc. TM-1

To elucidate the pattern of transmission of chromosome 10G^a^ in successive backcross generations, we observed the frequencies and incidences of chromosome 10G^a^ in three generations from the BC_2_ to BC_4_. The results indicated that the frequencies and incidences of chromosome 10G^a^ decreased slightly from BC_2_ to BC_4_, while the frequencies of chromosome 10G^a^ inheritance were as high as 100 % when the 10G^a^ addition lines were self-pollinated at each BC generation (Table 4).

**Table 4:** Transmission rates of the alien chromosome 10G^a^ in generations BC_2_ to BC_4_

Lineage	No. of progenies from MAAL-10G^a^ self-pollinated		Total
	2*n* (%)	2*n* + 1 (%)	
BC_2_F_1_	3 (6.4)	44 (93.6)	47
BC_2_F_2_	0 (0.00)	8 (100.0)	8
BC_3_F_1_	10 (12.2)	72 (87.8)	82
BC_3_F_2_	0 (0.00)	18 (100.0)	18
BC_4_F_1_	27 (14.7)	157 (85.3)	184
BC_4_F_2_	0 (0.00)	30 (100.0)	30

### Morphological traits of MAALs

**Fig. 4:**
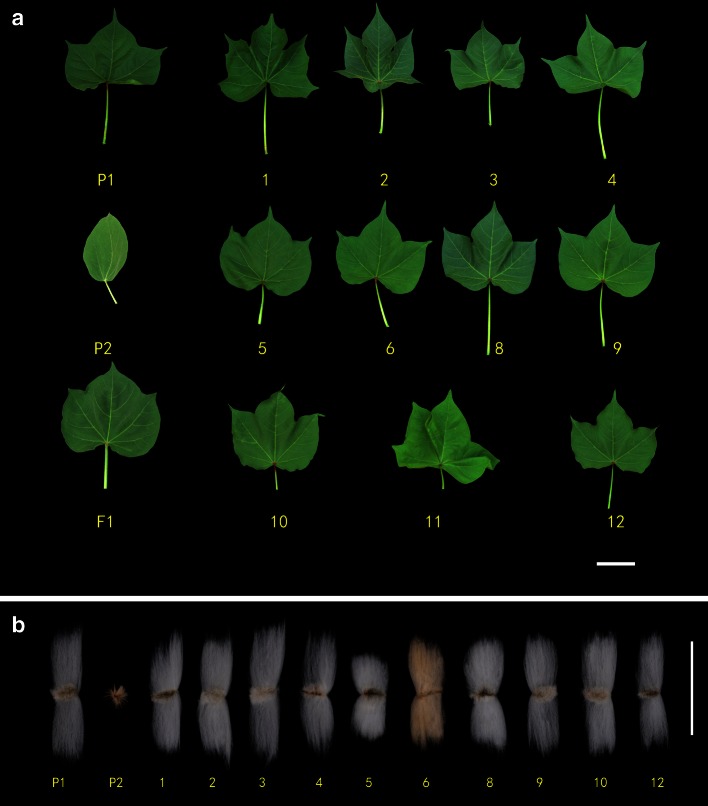
**a**–**b** Leaf shapes and fiber traits of MAALs of *G. australe* individual chromosomes in *G. hirsutum*. *P1* *G. hirsutum*, *P2* *G. australe*, *F*_1_ the hexaploid of *G. hirsutum* and *G. australe*. *1–6* and *8–12* are fibers from plants possessing single different individual chromosomes from *G. australe*, corresponding to 1G^a^ to 6G^a^, and 8G^a^ to 12G^a^ (but not 7G^a^ and 13G^a^). The absence of number 11 in B is because there were no bolls set due to the complete sterility of MAAL-11G^a^. *Scale bar* 50 mm

**Fig. 5:**
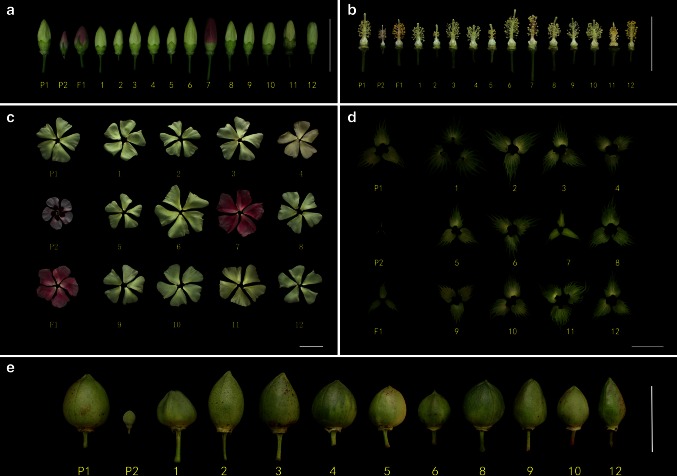
**a**–**e** Flower traits are shown for MAALs of *G. australe* individual chromosomes in *G. hirsutum*. **a**–**d** *P1* *G. hirsutum*, *P2* *G. australe*, *F*_1_ the hexaploid of *G. hirsutum* and *G. australe*. *1–6* and *8–12* are plants that carry a single different individual chromosome from *G. australe*, corresponding to 1G^a^ to 6G^a^ and 8G^a^ to 12G^a^; 7 is the triple alien addition line of chromosomes 7G^a^, 8G^a^, and 10G^a^. **e** Except for the absence of 7G^a^, 11G^a^, and 13G^a^, the numbers are the same as in **a**–**d**. *Scale bar* 50 mm

**Fig. 6:**
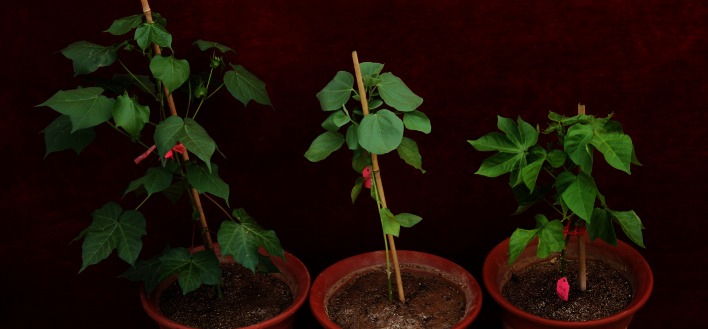
Whole plant traits of *G. hirsutum* acc. TM-1 (*left*), *G. australe* (*center*), and MAAL-11G^a^ (*right*)

Most of the MAALs exhibited slow growth rates compared to the *G. hirsutum* accession TM-1. In addition, their morphological traits differed from one another, and they also differed from the normal diploid *G. australe* in fundamental morphological features such as growth habit, height, shape and length of leaves, and pollen fertility (Tables 5, 6; Figs. 4a–b, 5, 6). In the present experiment, all the MAALs isolated had slow growth rates except MAAL-9G^a^ and MAAL-4G^a^, which were as vigorous as *G. hirsutum* TM-1.

**Table 5:** Morphological characters in eleven MAALs

Trait	P1	P2	Monosomic alien addition line		
	TM-1	*G. australe*	1G	2G	3G
Petal color	Creamy	Mauve	Creamy	Creamy	Creamy
Pollen color	Creamy	Gray	Creamy	Creamy	Creamy
Petal length (cm)	4.44 ± 0.14	3.46 ± 0.07	4.41 ± 0.10	3.77 ± 0.13	4.68 ± 0.16
Petal width (cm)	4.15 ± 0.11	3.35 ± 0.08	4.44 ± 0.15	3.90 ± 0.15	4.00 ± 0.11
Style length (cm)	3.50 ± 0.11	2.12 ± 0.04	2.80 ± 0.05	2.66 ± 0.10	3.16 ± 0.17
Stigma length (cm)	1.00 ± 0.07	0.56 ± 0.05	0.80 ± 0.05	0.81 ± 0.04	0.87 ± 0.07
Another number	125.20 ± 2.39	101.40 ± 2.41	111.40 ± 3.97	65.50 ± 3.82	105.40 ± 3.65
Pedicel length (cm)	1.52 ± 0.08	0.32 ± 0.04	1.54 ± 0.11	0.42 ± 0.12	1.02 ± 0.08
Calyx teeth length (cm)	0.24 ± 0.08	0.74 ± 0.08	0.36 ± 0.15	0.26 ± 0.10	0.22 ± 0.07
Leaf length (cm)	12.47 ± 0.70	8.40 ± 0.36	14.00 ± 0.87	13.83 ± 0.79	13.93 ± 0.55
Leaf width (cm)	15.57 ± 0.86	7.93 ± 0.93	20.37 ± 2.23	17.17 ± 0.97	13.43 ± 0.81
Petiole length (cm)	11.30 ± 1.91	3.40 ± 0.95	13.60 ± 4.01	9.63 ± 2.12	8.40 ± 1.51

Trait	Monosomic alien addition line				
	4G	5G	6G	8G	9G
Petal color	Creamy	Creamy	Creamy	Creamy	Creamy
Pollen color	Creamy	Creamy	Creamy	Creamy	Creamy
Petal length (cm)	4.14 ± 0.10	3.78 ± 0.16	5.04 ± 0.15	4.21 ± 0.12	4.28 ± 0.12
Petal width (cm)	3.78 ± 0.16	3.76 ± 0.16	4.78 ± 0.13	3.79 ± 0.12	3.99 ± 0.11
Style length (cm)	2.67 ± 0.13	2.76 ± 0.18	3.62 ± 0.08	3.42 ± 0.19	3.22 ± 0.20
Stigma length (cm)	0.82 ± 0.08	0.80 ± 0.04	1.28 ± 0.08	0.96 ± 0.09	0.87 ± 0.04
Another number	125.60 ± 2.41	70.40 ± 3.91	122.60 ± 5.73	114.60 ± 2.70	112.00 ± 2.55
Pedicel length (cm)	0.58 ± 0.08	0.56 ± 0.09	1.68 ± 0.08	1.54 ± 0.05	1.00 ± 0.20
Calyx teeth length (cm)	0.24 ± 0.08	0.27 ± 0.12	0.22 ± 0.09	0.28 ± 0.10	0.24 ± 0.10
Leaf length (cm)	8.07 ± 0.40	11.23 ± 0.55	8.30 ± 0.26	12.10 ± 0.53	9.07 ± 0.42
Leaf width (cm)	9.53 ± 0.56	13.00 ± 1.50	8.97 ± 0.65	13.77 ± 0.51	11.20 ± 0.98
Petiole length (cm)	14.60 ± 0.12	8.42 ± 1.72	9.67 ± 0.60	13.60 ± 0.40	13.01 ± 1.33

Trait	Monosomic alien addition line				
	10G	11G	12G		
Petal color	Creamy	Creamy	Creamy		
Pollen color	Creamy	Creamy	Light yellow		
Petal length (cm)	4.78 ± 0.11	4.76 ± 0.15	4.90 ± 0.18		
Petal width (cm)	4.36 ± 0.09	4.40 ± 0.10	4.04 ± 0.17		
Style length (cm)	3.06 ± 0.17	3.11 ± 0.14	3.00 ± 0.16		
Stigma length (cm)	0.86 ± 0.05	0.87 ± 0.06	0.71 ± 0.09		
Another number	121.40 ± 3.13	82.00 ± 3.40	102.80 ± 1.64		
Pedicel length (cm)	1.08 ± 0.08	1.11 ± 0.09	1.00 ± 0.12		
Calyx teeth length (cm)	0.18 ± 0.09	0.49 ± 0.10	0.26 ± 0.10		
Leaf length (cm)	13.83 ± 0.71	13.20 ± 0.65	9.43 ± 0.75		
Leaf width (cm)	15.73 ± 0.81	17.10 ± 0.84	11.50 ± 0.50		
Petiole length (cm)	7.01 ± 2.03	6.02 ± 1.64	9.42 ± 0.91

**Table 6:** The unique traits of the monosomic alien addition lines

Alien chromosome added	Unique traits
1G^a^	Oblate boll and grooved leaves with irregularly serrate margins
2G^a^	Small flowers with few anthers
3G^a^	Very high lint percentage and normal fiber length
4G^a^	Normal fiber length and normal plant shape
5G^a^	Small bracts and flowers. Compact plant type and short fibers ca. 25 mm in length
6G^a^	Brown fibers
7G^a^	Pink flowers and hard stems
8G^a^	Dark green leaf with high concentration of chlorophyll *a* and chlorophyll *b*
9G^a^	Small bracts and small flowers
10G^a^	High rate of alien chromosome transmission
11G^a^	Completely sterile
12G^a^	Yellow anthers

MAAL-1G^a^ plant leaves were observed to be grooved with irregularly serrate margins (Fig. 4a), the leaf sizes were larger than those of TM-1, and its bract calyx had more and longer calyx teeth (Fig. 5d, Table 5). The plants grew slowly.

MAAL-2G^a^ plants had the lowest number of anthers among the 11 MAALs (Tables 5, 6). The flowers with shorter pedicels were significantly smaller than observed in the TM-1 plants. The leaf sizes were slightly larger as well, and the plants grew slowly.

MAAL-3 G^a^ plants produced normal fiber lengths and had a very high lint percentage due to the extremely low seed index. The flower sizes were similar to those of the TM-1 plants (Table 5).

MAAL-4G^a^ plants had fewer hairs on the stems and produced round bolls with larger seeds; the seed index was 13.17 g, which was a significant increase of 26.27 % over TM-1 (10.43 g). The flowers and leaves were slightly smaller than that of TM-1 (Table 5).

MAAL-5G^a^ plants displayed a compact plant type with shorter fibers (Fig. 4b), shorter pedicels and petioles, shorter fruit branches, and smaller flowers (Fig. 5a–e) than those of TM-1. These plants grew slowly. MAAL-6G^a^ plants were easily distinguishable by its brown-colored fibers and the fact that it had the largest flowers among the 11 MAALs (Figs. 4b, 5a–c). The leaves were smaller than those of TM-1 plants and these plants grew slowly (Table 5). MAAL-7G^a^ could be identified by the pink flower; even though this chromosome has not been isolated in a single MAAL (Fig. 5a–c), as neither the monosomic alien addition lines that carried 8G^a^ or 10G^a^ had a pink flower. Thus, based on the morphological traits, the triple monosomic alien addition line should carry chromosomes 7G^a^, 8G^a^, and 10G^a^ simultaneously.

MAAL-8G^a^ plants were easily distinguishable by its dark green leaves; the chlorophyll *a* and chlorophyll *b* contents were as high as 1065.39 and 417.01 mg/g, respectively, significantly higher than the values for TM-1, which were 736.1 and 281.14 mg/g (Table 7). These plants grew slowly.

**Table 7:** Photosynthetic pigment contents in *G. hirsutum* and three MAALs

Line	Chlorophyll *a*	Chlorophyll *b*	Carotenoids
TM-1	728.82 ± 8.05	288.29 ± 14.57	65.79 ± 1.14
MAAL-8G^a^ (1)	1,118.35 ± 26.28**	414.75 ± 22.84**	101.93 ± 16.33**
MAAL-8G^a^ (2)	1,109.77 ± 31.15**	409.09 ± 26.87**	106.03 ± 19.81**
MAAL-8G^a^ (3)	1,071.28 ± 6.40**	402.18 ± 23.39**	102.35 ± 1.14**

** Significance at the 1 % level

MAAL-9G^a^ plants had longer fruit-branch nodes than those of TM-1 plants, and they were pagoda-like in shape. The leaves were smaller than those of TM-1 plants (Table 5).

MAAL-10G^a^ plants grew slowly and its alien chromosome was transmitted at the high rate of 96.5 %. The flowers were slightly larger than those of the TM-1 plants, and the leaves were similar in size to that of the TM-1 plant (Table 5).

The MAAL-11G^a^ plant had large light-green leaves with folded lobes, and showed complete male and female sterility—the anthers did not disperse pollen, and no bolls were set when it was used as a maternal parent (Figs. 4a, 6). Chromosome 11G^a^ seems to carry genes that cause sterility. The flowers were slightly larger than those of the TM-1 plant, while the leaves were larger (Table 5). This plant grew very slowly.

MAAL-12G^a^ plants were highly distinguishable because of their small, cone-shape bolls (Fig. 5e). The flowers were slightly larger than those of the TM-1 plants, while the leaves were smaller (Table 5). These plants also grew slowly.

## Discussion

### Challenges in developing hirsutum wild species alien chromosome addition lines

Monosomic alien addition lines (MAALs), which carry an additional chromosome from a related species through interspecific/intergenomic hybridization, have become powerful tools for genetic and genomic research. These MAALs can also be used to improve the process of genetic introgression of desirable genes from donor species into recipient species through the production of chromosome substitution and translocation lines. However, the development of cotton MAALs is an extremely laborious and frustrating process using conventional cytogenetic techniques, because the chromosomes of cotton are numerous (2*n* = 52) and are too small for reliable identification and characterization in mitotic and meiotic cells (genome size is approximately 2.5 Gb). At present, incomplete sets of MAALs from several wild diploid *Gossypium* species have been developed in *G. hirsutum*; however, no complete set of MAALs has been reported, even though many efforts have been made since the early 1980s (Hau 1981; Rooney and Stelly 1991; Mergeai 1992). With the advent of molecular markers and genomic in situ hybridization (GISH) techniques utilized in cotton, this situation has changed drastically. Through the combination of SSR markers and GISH analysis, Ahoton et al. (2003) isolated six of the possible 13 MAALs carrying *G. australe* chromosomes in a *G. hirsutum* background. Subsequently, Sarr et al. (2011) also isolated five new MAALs of *G. australe* in *G. hirsutum* using the methods of Ahoton et al. (2003). Zhou et al. (2004) also isolated two MAALs of *G. somalense* chromosomes in *G. hirsutum* using random amplified polymorphic DNA (RAPD) and classical cytogenetic analysis. In this study, we have developed the first complete set of alien chromosome addition lines in cotton; 11 of these lines are monosomic additions, with chromosomes 7G^a^ and 13G^a^ present in multiple addition lines. GISH allowed us to show that one or more alien chromosomes were present, and chromosome-specific markers allowed us to identify the alien chromosomes by genotyping in the cultivated cotton background. Our results indicated that the combination of SSR markers and GISH analysis can facilitate the efficient establishment of monosomic alien addition lines in cotton.

During the isolation of cotton MAALs, we found that most of the alien (*G. australe*) chromosomes were transmitted through the maternal parent at a very low rate, while one chromosome, 10G^a^, was transmitted maternally at an extremely high frequency. Differences in the transmission rates of individual alien chromosomes in monosomic alien addition lines have been reported previously in tomato by Ali et al. (2001). Although there is no clear explanation for the phenomenon, Ali et al. (2001) believed that different genetic backgrounds might influence the transmission of alien chromosomes, so they proposed that the selection of proper backcross populations was important. In our study, alien chromosome transmission biases and different transmission rates were observed, as have been previously reported in cotton (Lopez-Lavalle and Brubaker 2007; Rooney and Stelly 1991) and tomato (Ali et al. 2001). To further facilitate the elucidation of the genetic roles for each alien chromosome in upland cotton in the future, the standard genetic line TM-1 was used as the sole recurrent parent to consecutively backcross to the progeny of *G. hirsutum* and *G. australe*, leading to a uniform genetic background in the MAALs. But the genetic background of TM-1 may have differential effects on the transmission of individual chromosomes of *G. australe* in *G. hirsutum* during successive backcrossing; for example, no single chromosome addition lines for 7G^a^ and 13G^a^ were isolated, because single 7G^a^ and 13G^a^ chromosomes were either not transmitted to the progeny or were always transmitted with other alien chromosomes in the TM-1 background. If so, the isolation of monosomic alien addition lines for chromosomes with low transmission rates will necessitate using different parental lines during backcrossing. For isolation of MAALs of 7G^a^ and 13G^a^ to complete the set of MAALs of *G. australe* in *G. hirsutum*, it will be necessary to test alternative recurrent parents in the future.

Another question is whether homoeologous recombination has occurred between chromosomes of *G. australe* and *G. hirsutum* during the development of the MAALs. Theoretically, if homoeologous chromosomes of *G. australe* and *G. hirsutum* were to pair at meiosis, recombination could possibly occur. Previous cytological investigations on chromosomal configuration in pollen mother cells at meiosis in a triploid F_1_ showed that there was a very low rate of chromosome pairing between AADD and G-genome chromosomes (Liang et al. 1997), indicating the distant relationship that exists between chromosomes from the AADD and G genomes. In addition, cytological investigation into the behavior of monosomic alien chromosomes showed that very little or no pairing with AD chromosomes was observed in this study (Fig. 3j–l), implying that there is very little or no homoeologous recombination between them. However, homoeologous recombination in the aneuploid progeny still cannot be completely ruled out. Furthermore, it could be speculated that recombination is more likely to happen between Ah and G chromosomes than between Dh and G chromosomes due to G being phylogenetically closer to Ah than to Dh. These hypotheses are based on the results of GISH (Guan et al. 2008) and molecular phylogenetic evolution analyses for the *Gossypium* genus by Wendel and Cronn (2003).

**Fig. 7:**
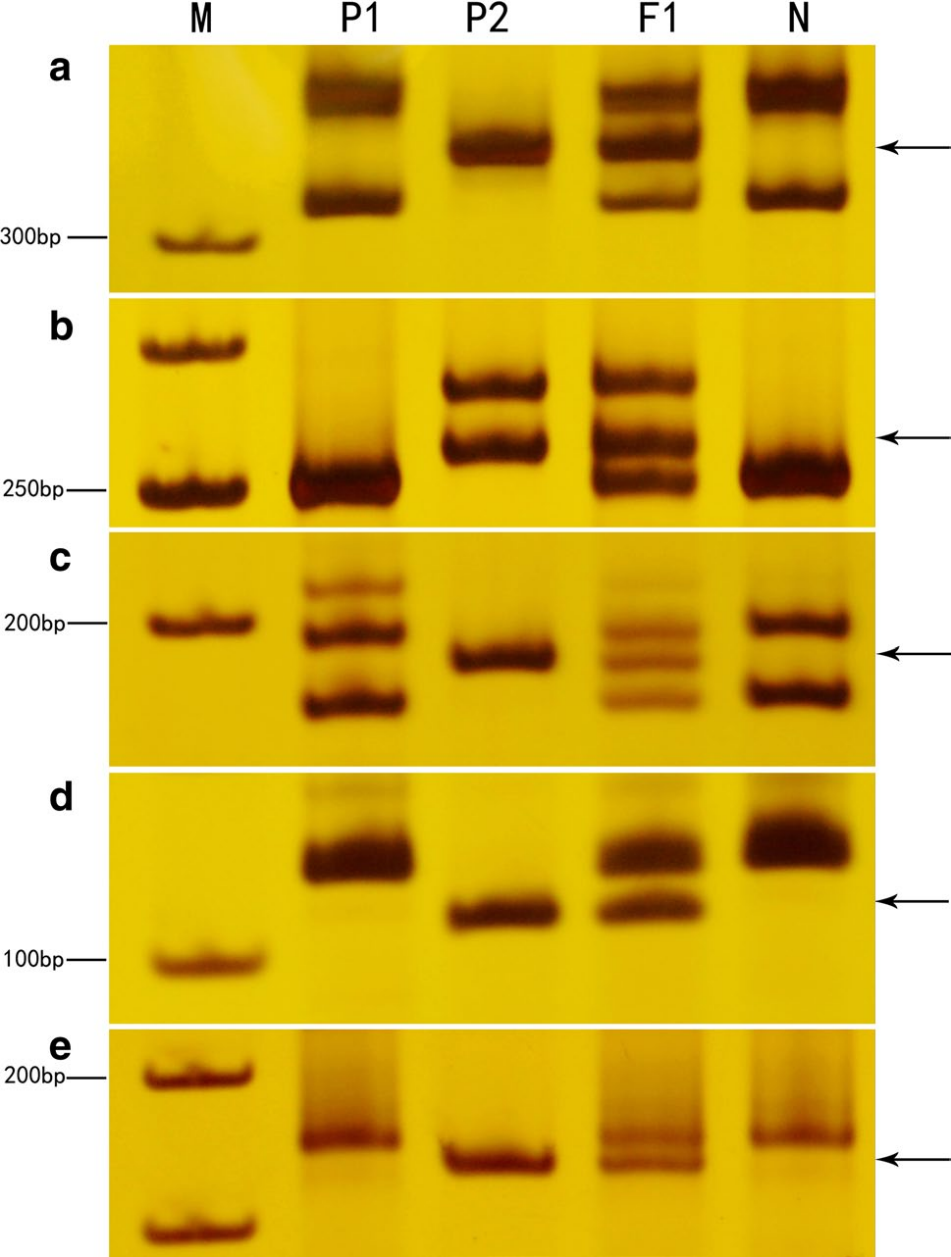
**a**–**e** *G. australe*-*specific* DNA fragments detected using SSR markers during backcrossing were deleted in some MAALs in *G. hirsutum*. **a**–**e** Deleted *G. australe*-specific amplicons were detected by individual chromosome-specific SSR primers for NAU6728 (2G^a^), NAU5486 (5G^a^), MUSS266 (10G^a^), BNL193 (13G^a^), and CIR414 (11G^a^), respectively. *P1* *G. hirsutum*, *P2* *G. australe*. *F*_1_ the hexaploid of *G. hirsutum* and *G. australe*. *N* indicates MAAL-2G^a^ (**a**), MAAL-5G^a^ (**b**), MAAL-10G^a^ (**c**), MAAL-13G^a^ (**d**), and MAAL-11G^a^ (**e**). *M* is the molecular size marker (50 bp ladder). *Arrows* indicate the deleted *G. australe* chromosome-specific markers

If homoeologous recombination were to occur, it could damage the integrity of the alien chromosome, and such MAALs should be identified. However, it is very difficult with present methods to detect recombination. The GISH technique cannot easily detect recombinant segments in the recipient genome, because cotton chromosomes are very small and there is very little or no repetitive DNA present in the euchromatic regions (Ali et al. 2001). Molecular markers could be used to detect recombinant DNA segments if they were polymorphic. For instance, if a plant was identified by GISH as being a cytological euploid without alien chromosomes, but species-specific markers from the donor parent were present, the occurrence of homoeologous recombination would be confirmed. But no such plant has been found in our study. Thus, it can be concluded that very little, if any, homoeologous recombination occurred during the development of MAALs. Conversely, several MAALs were found to be missing a few species-specific markers (Table 1; Fig. 7, Fig. 1S), which might result from the elimination of some DNA fragments during backcrossing or polyploidization (Fig. 1S). This phenomenon has been reported previously in cotton and other crops (Jiang et al. 2000; Ozkan et al. 2001; Shaked et al. 2001; Skalicka et al. 2005).

### Strategies for developing cotton cultivars with the glandless-seed and glanded-plant traits

The genus *Gossypium* is a globally important crop that is used to produce textiles, oil, and protein. Gossypol in cultivated cottonseed, however, is toxic to humans and non-ruminant animals but highly related to insect resistance (Bottger et al. 1964). Due to gossypol mainly existed in the cotton pigment gland, thus, breeding a high-yielding “glandless-seed” and “glanded-plant” cultivar has become an area of interest for researchers. Efforts have been made to eliminate gossypol from cottonseeds while keeping it in foliar vegetative tissues. Since Muramoto first synthesized a fertile hexaploid of (*G. hirsutum* × *G. sturtianum*) F_1_ (Australian wild species, C genome), three Australian wild species [*G. bickii*, *G. australe* (G genome) and *G. sturtianum*] have been used in interspecific breeding through both the hexaploid and tetraploid pathways. In the hexaploid pathway, tetraploid cotton (2*n* = AADD = 52) was crossed with diploid wild-type species (2*n* = CC or GG = 26) to produce a triploid F_1_. Then, the obtained triploid F_1_ was chromosome doubled and the hexaploid was subsequently developed. Through the hexaploid pathway, breeding efforts for the introgression of glanded-plant and glandless-seed trait were made (Muramoto 1969; Dilday 1986; Altman et al. 1987). He and Sun (1994) studied the hybrid F_1_ between glandless upland cotton and *G. bickii*. Liang et al. (1997) developed a new red-flowered germplasm line of cotton selected from a hybrid of *Gossypium hirsutum* × *G. bickii*. These have been used in hybrid cotton (Wang and Wang 2008).

He et al. (2000) analyzed the inheritance of the petal color genes introgressed from *G. bickii*. Using the tetraploid pathway, diploid cotton (2*n* = AA or DD = 26) was crossed with diploid wild-type species (2*n* = CC or GG = 26) to produce a diploid F_1_. The obtained diploid F_1_ was then chromosome doubled to develop the tetraploid. For example, Mergeai (1992) developed an amphidiploid F_1_ of *G. arboreum* × *G. sturtianum* with glandless-seed and glanded-plant traits, which were sequentially used as bridge material for the transfer of the target trait into upland cotton (Bi et al. 1998; Bi et al. 1998, 1999a, b; Ahoton et al. 2003). Kulkarni et al. (2002) carried out studies on the interspecific hybridization of *G. australe* and *G. herbaceum*. Zhang et al. (1994) synthesized allotetraploid cotton with an (AG) complex chromosome set, which has since been used in breeding programs (Zhu et al. 2004). However, the “glandless-seed” and “glanded-plant” cultivar has not been developed at present. The glandless-seed, glanded-plant trait is present only in some Australian wild diploid cotton species with C and G genomes, which are phylogenetically distant from the cultivated upland cotton, *G. hirsutum*. The formation of gossypol glands in upland cotton is controlled by two main alleles, Gl_2_ and Gl_3_ (McMichael 1960). Genetic analysis by Zhu et al. (2004) demonstrated that “glandless-seed” and “glanded-plant” trait from *G. bickii*, another Australian wild G-genome species, is controlled by a gene located at the *Gl*_*2*_ locus, which has been temporarily named *Gl*_*2*_^*b*^. This gene, *Gl*_*2*_^*b*^, is dominant to upland cotton pigment gland alleles *Gl*_*2*_ and *gl*_*2*_, but is recessive and epistatic to another glanded gene *Gl*_*3*_. Based on these results, it is presumed that only the MAAL with *Gl*_*2*_^*b*^ *Gl*_*2*_ *Gl*_*2*_ *gl*_*3*_ *gl*_*3*_ or *Gl*_*2*_^*b*^ *gl*_*2*_ *gl*_*2*_ *gl*_*3*_ *gl*_*3*_ will show the glandless-seed, glanded-plant phenotype. Using normal upland cotton with the dominant gene *Gl*_*3*_ as the recipient parent, it will be impossible to obtain a MAAL with the glandless-seed, glanded-plant trait, since the genotype of the MAAL is *Gl*_*2*_^*b*^ *Gl*_*2*_ *Gl*_*2*_ *Gl*_*3*_ *Gl*_*3*_. This also could explain why no MAAL with this trait was detected in our study. Thus, to develop a MAAL with glandless-seed, glanded-plant trait, the recipient upland cotton parent used should carry the recessive *gl*_*3*_ gene. To largely eliminate the role of *Gl*_*3*_ in conferring gossypol synthesis, the ideal cotton genotype would be *Gl*_*2*_^*b*^ *Gl*_*2*_^*b*^ *Gl*_*2*_ *Gl*_*2*_ without *Gl*_*3*_ *Gl*_*3*_. The only way to obtain a “glandless-seed” and “glanded-plant” cultivar with the *Gl*_*2*_^*b*^ *Gl*_*2*_^*b*^ *Gl*_*2*_ *Gl*_*2*_ genotype would be to use radiation to induce chromosomal translocation between the homoeologous chromosomes 12D_t_ in *G. hirsutum* and 12G^a^ in *G. australe*, since very little or no homoeologous recombination occurs under natural conditions.

## Electronic supplementary material

Below is the link to the electronic supplementary material.

Tables 1S–4S (DOC 87 kb)

Figure 1S The set of putative *G. australe* chromosome-specific SSR markers that were used to screen for polymorphisms. Markers are based on the backbone map of the D_t_-subgenome of tetraploid cotton constructed using the BC_1_ population of (*G. hirsutum* × *G. barbadense*) × *G. hirsutum* (Guo et al. 2007). Note: Markers in red were eliminated in the corresponding monosomic alien addition lines during backcrossing (DOC 177 kb)
