# Supplementary material for: Construction of a complete set of alien chromosome addition lines from Gossypium australe in Gossypium hirsutum: morphological, cytological, and genotypic characterization
Source: Theor Appl Genet. 2014 Feb 20;127(5):1105–21. doi: 10.1007/s00122-014-2283-1 (PMC3997835; doi:10.1007/s00122-014-2283-1)
Supplement: Supplementary file 1 — Tables 1S–4S (DOC 87 kb) [file 122_2014_2283_MOESM1_ESM.doc]

**Table 1S.** Incidence of alien (*G. australe*) chromosomes in *G. hirsutum* x *G. australe* in the BC_2_ generation

| Chromosome number | 1G | 2G | 3G | 4G | 5G | 6G | 7G | 8G | 9G | 10G | 11G | 12G | 13G | No. of the individuals |
| --- | --- | --- | --- | --- | --- | --- | --- | --- | --- | --- | --- | --- | --- | --- |
| 52+0 | 0 | 0 | 0 | 0 | 0 | 0 | 0 | 0 | 0 | 0 | 0 | 0 | 0 | 0 |
| 52+1 | 0 | 2 | 0 | 0 | 2 | 0 | 0 | 0 | 0 | 2 | 2 | 0 | 0 | 8 |
| 52+2 | 0 | 1 | 1 | 1 | 1 | 2 | 0 | 1 | 0 | 7 | 0 | 3 | 1 | 9 |
| 52+3 | 1 | 2 | 3 | 2 | 3 | 2 | 1 | 5 | 1 | 10 | 1 | 2 | 0 | 11 |
| 52+4 | 4 | 2 | 4 | 3 | 2 | 1 | 0 | 4 | 1 | 10 | 1 | 5 | 3 | 10 |
| 52+5 | 0 | 1 | 5 | 5 | 4 | 2 | 2 | 4 | 0 | 8 | 2 | 5 | 2 | 8 |
| 52+6 | 2 | 5 | 3 | 3 | 5 | 1 | 3 | 5 | 2 | 7 | 5 | 5 | 2 | 8 |
| 52+7 | 2 | 4 | 3 | 1 | 3 | 3 | 1 | 4 | 0 | 4 | 2 | 1 | 0 | 4 |
| 52+8 | 2 | 1 | 3 | 3 | 3 | 1 | 0 | 3 | 1 | 3 | 1 | 1 | 2 | 3 |
| 52+9 | 1 | 1 | 1 | 1 | 0 | 0 | 0 | 1 | 1 | 1 | 1 | 1 | 0 | 1 |
| 52+10 | 1 | 1 | 1 | 1 | 1 | 1 | 0 | 0 | 1 | 1 | 0 | 1 | 1 | 1 |
| sum | 13 | 20 | 24 | 20 | 24 | 13 | 7 | 27 | 7 | 53 | 15 | 24 | 11 | 63 |
| Incidence (%) | 20.63 | 31.75 | 38.10 | 31.75 | 38.10 | 20.63 | 11.11 | 42.86 | 11.11 | 84.13 | 23.81 | 38.10 | 17.46 |  |
| Monosomic addition (%) | 0.00 | 25.00 | 0.00 | 0.00 | 25.00 | 0.00 | 0.00 | 0.00 | 0.00 | 25.00 | 25.00 | 0.00 | 0.00 |  |

**Table 2S.** Incidence of alien (*G. australe*) chromosomes in *G. hirsutum* x *G. australe* in the BC_3_ generation

| Chromosome number | 1G | 2G | | 3G | | 4G | | 5G | | 6G | | 7G | | 8G | | 9G | | 10G | | 11G | | 12G | | 13G | | No. of the individuals |  |
| --- | --- | --- | --- | --- | --- | --- | --- | --- | --- | --- | --- | --- | --- | --- | --- | --- | --- | --- | --- | --- | --- | --- | --- | --- | --- | --- | --- |
| 52+0 | 0 | 0 | | 0 | | 0 | | 0 | | 0 | | 0 | | 0 | | 0 | | 0 | | 0 | | 0 | | 0 | | 17 |  |
| 52+1 | 1 | 0 | | 1 | | 0 | | 0 | | 3 | | 0 | | 9 | | 0 | | 51 | | 0 | | 1 | | 0 | | 66 |  |
| 52+2 | 0 | 2 | | 8 | | 2 | | 4 | | 1 | | 0 | | 7 | | 2 | | 31 | | 1 | | 13 | | 2 | | 36 |  |
| 52+3 | 1 | 1 | | 10 | | 2 | | 2 | | 5 | | 0 | | 4 | | 2 | | 19 | | 0 | | 11 | | 3 | | 20 |  |
| 52+4 | 3 | 7 | | 6 | | 4 | | 3 | | 8 | | 2 | | 5 | | 2 | | 20 | | 2 | | 11 | | 7 | | 20 |  |
| 52+5 | 0 | 4 | | 3 | | 0 | | 1 | | 1 | | 1 | | 4 | | 1 | | 5 | | 0 | | 4 | | 1 | | 5 |  |
| sum | 5 | 14 | | 28 | | 8 | | 10 | | 18 | | 3 | | 29 | | 7 | | 126 | | 3 | | 40 | | 13 | | 147 |  |
| Incidence (%) | 3.40 | 9.52 | 19.05 | | 5.44 | | 6.80 | | 12.24 | | 2.04 | | 19.73 | | 4.76 | | 85.71 | | 2.04 | | 27.21 | | 8.84 | |  | | |
| Monosomic addition (%) | 1.52 | 0.00 | 1.52 | | 0.00 | | 0.00 | | 4.55 | | 0.00 | | 13.64 | | 0.00 | | 77.27 | | 0.00 | | 1.52 | | 0.00 | |  | | |

**Table 3S.** Incidence of alien (*G. australe*) chromosomes in *G. hirsutum* x *G. australe* in the BC_4_ generation

| Chromosome number | 1G | 2G | 3G | 4G | 5G | 6G | 7G | 8G | 9G | 10G | 11G | 12G | 13G | No. of the individuals |
| --- | --- | --- | --- | --- | --- | --- | --- | --- | --- | --- | --- | --- | --- | --- |
| 52+0 | 0 | 0 | 0 | 0 | 0 | 0 | 0 | 0 | 0 | 0 | 0 | 0 | 0 | 18 |
| 52+1 | 0 | 0 | 5 | 0 | 1 | 2 | 0 | 3 | 1 | 122 | 0 | 2 | 0 | 136 |
| 52+2 | 0 | 2 | 14 | 3 | 0 | 5 | 0 | 4 | 2 | 60 | 0 | 38 | 2 | 65 |
| 52+3 | 0 | 2 | 9 | 1 | 1 | 2 | 0 | 2 | 0 | 16 | 0 | 10 | 6 | 16 |
| 52+4 | 0 | 0 | 1 | 0 | 1 | 0 | 0 | 1 | 0 | 2 | 0 | 2 | 1 | 2 |
| sum | 0 | 4 | 29 | 4 | 3 | 9 | 0 | 10 | 3 | 200 | 0 | 52 | 9 | 219 |
| Incidence (%) | 0.00 | 1.83 | 13.24 | 1.83 | 1.37 | 4.11 | 0.00 | 4.57 | 1.37 | 91.32 | 0.00 | 23.74 | 4.11 |  |
| Monosomic addition (%) | 0.00 | 0.00 | 3.68 | 0.00 | 0.74 | 1.47 | 0.00 | 2.21 | 0.74 | 89.71 | 0.00 | 1.47 | 0.00 |  |

**Table 4S.** Incidence of alien (*G. australe*) chromosomes in *G. hirsutum* x *G. australe* in the BC_5_ generation

| Chromosome number | 1G | 2G | 3G | 4G | 5G | 6G | 7G | 8G | 9G | 10G | 11G | 12G | 13G | No. of the individuals |
| --- | --- | --- | --- | --- | --- | --- | --- | --- | --- | --- | --- | --- | --- | --- |
| 52+0 | 0 | 0 | 0 | 0 | 0 | 0 | 0 | 0 | 0 | 0 | 0 | 0 | 0 | 15 |
| 52+1 | 0 | 0 | 1 | 1 | 1 | 0 | 0 | 0 | 0 | 8 | 0 | 0 | 0 | 11 |
| 52+2 | 0 | 0 | 0 | 5 | 0 | 0 | 0 | 0 | 0 | 5 | 0 | 0 | 0 | 5 |
| 52+3 | 0 | 0 | 0 | 0 | 1 | 0 | 0 | 0 | 0 | 1 | 0 | 0 | 1 | 1 |
| sum | 0 | 0 | 1 | 6 | 2 | 0 | 0 | 0 | 0 | 14 | 0 | 0 | 1 | 17 |
| Incidence (%) | 0.00 | 0.00 | 5.88 | 35.29 | 11.76 | 0.00 | 0.00 | 0.00 | 0.00 | 82.35 | 0.00 | 0.00 | 5.88 |  |
| Monosomic addition (%) | 0.00 | 0.00 | 9.09 | 9.09 | 9.09 | 0.00 | 0.00 | 0.00 | 0.00 | 72.73 | 0.00 | 0.00 | 0.00 |  |
