# Supplementary material for: Construction of a complete set of alien chromosome addition lines from Gossypium australe in Gossypium hirsutum: morphological, cytological, and genotypic characterization
Source: Theor Appl Genet. 2014 Feb 20;127(5):1105–21. doi: 10.1007/s00122-014-2283-1 (PMC3997835; doi:10.1007/s00122-014-2283-1)
Supplement: Supplementary file 2 — Figure 1S The set of putative G. australe chromosome-specific SSR markers that were used to screen for polymorphisms. Markers are based on the backbone map of the Dt-subgenome of tetraploid cotton constructed using the BC1 population of (G. hirsutum × G. barbadense) × G. hirsutum (Guo et al. 2007). Note: Markers in red were eliminated in the corresponding monosomic alien addition lines during backcrossing (DOC 177 kb) [file 122_2014_2283_MOESM2_ESM.doc]

**Figure 1S** The set of putative *G. australe* chromosome-specific SSR markers that were used to screen for polymorphisms. Markers are based on the backbone map of the Dt subgenome of tetraploid cotton constructed using the BC_1_ population of (*G. hirsutum* × *G. barbadense*) × *G. hirsutum* (Guo et al. 2007).

Note: Markers in red were eliminated in the corresponding monosomic alien addition lines during backcrossing.
